# Hepigenetics: A Review of Epigenetic Modulators and Potential Therapies in Hepatocellular Carcinoma

**DOI:** 10.1155/2020/9593254

**Published:** 2020-11-24

**Authors:** Mohamed H. Yousef, Hassan A. N. El-Fawal, Anwar Abdelnaser

**Affiliations:** ^1^Biotechnology Graduate Program, School of Science and Engineering, The American University in Cairo, Cairo, Egypt; ^2^Institute of Global Health and Human Ecology, School of Science and Engineering, The American University in Cairo, Cairo, Egypt

## Abstract

Hepatocellular carcinoma is the fifth most common cancer worldwide and the second most lethal, following lung cancer. Currently applied therapeutic practices rely on surgical resection, chemotherapy and radiotherapy, or a combination thereof. These treatment options are associated with extreme adversities, and risk/benefit ratios do not always work in patients' favor. Anomalies of the epigenome lie at the epicenter of aberrant molecular mechanisms by which the disease develops and progresses. Modulation of these anomalous events poses a promising prospect for alternative treatment options, with an abundance of felicitous results reported in recent years. Herein, the most recent epigenetic modulators in hepatocellular carcinoma are recapitulated on.

## 1. Introduction

Hepatocellular carcinoma (HCC) is a notoriously aggressive cancer with high global prevalence rates and is the next most common perpetrator of cancer-related death following pulmonary carcinomas, with annual mortality rates of the order of 800,000 deaths [1]. HCC develops in a backdrop of a chronic liver disease that ultimately results in liver fibrosis and cirrhosis, which are consequential HCC risk factors. Hepatitis C and B, aflatoxins, alcoholic liver disease, and nonalcoholic steatohepatitis are all commonly encountered chronic inflammatory hepatopathologies that predispose to HCC. Depending on the etiology, disparate molecular dysregulation patterns arise, all converging on promoting malignancy. The loss of cell cycle restraints, incapacity to senesce, and disarrayed apoptosis [2] are among such dysregulated mechanisms, which could well be the result of genetic as well as epigenetic alterations.

The epigenome constitutes heritable features of the genetic material out with the DNA sequence. Specific epigenetic patterns are important for the maintenance of cellular integrity and gene expression patterns associated with health. In this capacity, the epigenetic fingerprint functions to guarantee proper and timely expression of genetic information, and its alteration aggravates pernicious cellular changes, many of which predispose to cancer [3]. Herein, a compendium of the most recent work addressing epigenetic modulators in the context of HCC is presented.

### 1.1. What Is Epigenetics?

Epigenetics is a term that was first coined by Conrad Waddington, and it literally means “above genetics” [4]. It entails changes to cellular phenotypes, which are not dependent on alterations of the genetic code (DNA sequence). However, unanimity regarding the definition of epigenetics has thus far been elusive, and debates in this regard have been inconclusive at best [5].

As previously mentioned, the most recognized of epigenetic mechanisms involve chromatin remodeling. Chromatin is the macromolecule by virtue of which the genetic material can be packed inside cells' nuclei. It is composed of nucleosomes: DNA wound around histone protein octamers. In its compact form, the heterochromatin, the genetic material is relatively inaccessible for replication and the genes within are largely silent. The euchromatin on the other hand is a relaxed form of chromatin where the DNA is more accessible and genes are more or less actively expressed [5]. It can thus be easily concluded that regulation of chromatin condensation plays a role in regulating gene expression and the resulting phenotypes. Chromatin-modifying enzymes are key players in effecting such restructuring and subsequent modifications to DNA and the histone scaffolding on which it is wound.

CpG islands are clusters of CpG dinucleotides predominantly found in the promoter regions of genes. Generally, methylation of the 5-carbon in the cytosine of these CpG islands shields the promoter from the transcription machinery to the end result of a controlled gene expression. On the other hand, demethylation of these regions within gene promoters allows for the recruitment of the transcription machinery and the gene is essentially “on.” Such functionality is predominantly reserved for DNA methyltransferases. That being said, promoters containing CpG islands account for only 70% of the promoters in the genome. Interaction with the remaining 30% is orchestrated by modifications to the histone proteins, regulated—to a large extent—by histone deacetylases [5]. The disruption of these mechanisms can thus lead to aberrations in gene expression, which in many cases can initiate or promote oncogenesis. For example, the promoters of genes, which are normally turned off, are usually found hypomethylated in cancer.

### 1.2. Epigenetic Modulators

Options for epigenetic therapies in HCC can be enumerated as follows: inhibitors of DNA methyltransferases, regulators of histone methyltransferases, demethylases, acetyltransferases, and—most prominently—deacetylases. Another major class of epigenetic modulators is represented in noncoding RNAs. Below, the most eminent and clinically established classes are explored comprehensively to afford an encyclopedic overview of the current status of epigenetic recourse for HCC therapy. However, due to scarcity of data, several agents such tacedinaline, romidepsin, some helicases, and other enzymes viz. acireductone dioxygenase 1 are not discussed.

## 2. DNA Modifications

### 2.1. DNA Methyltransferases (DNMTs)

The implication of epigenetic changes in HCC, specifically aberrant patterns of DNA methylation, has recently been recognized as a primary contributor to disease onset and progression [6]. As a consequence of such epigenetic anomalies, key tumor suppressors may be silenced or oncogenes activated, resulting in the initiation of tumorigenesis. DNA methylation is mediated by a conserved class of catalytic proteins known as *DNA methyltransferases* (DNMTs). DNMTs are key players of the epigenome. DNMTs come in two primary categories, maintenance (DNMT1) and *de novo* DNMTs (DNMT3a and DNMT3b) [7]. Although the distinction is not absolute, it does hold contemporarily. *DNMT1*, *DNMT3a*, and *DNMT3b* function by catalyzing the transfer of a methyl group from S-adenosyl-L-methionine, the universal methyl donor to a 5′-cytosine on DNA [8]. Moreover, several other DNMTs do exist (such as *DNMT2* and *DNMTL*); however, they remain relatively undefined despite having demonstrated a role in HCC [9].

Despite the widely suggested distinction that *DNMT1* functions as the maintenance methyltransferase and *DNMT3a* and *DNMT3b* mediate *de novo* methylation (predominantly during embryonic development), the notion has been challenged as of late, with *DNMT1* recognized as a contributor to *de novo* methylation while maintenance functions are mediated by *DNMT3a* and *DNMT3b* in concert with *DNMT1* [10]. Notwithstanding the above-mentioned classification, these enzymes do not function individually and their interaction is crucial to the creation and maintenance of appropriate methylation patterns. The alteration of such coordination has in fact been associated with cancer development [11].

### 2.2. DNMT1

*DNMT1* is the most common subtype in adult cells [12]. Normally, *DNMT1* functions to maintain methylation patterns of CpG sites within promoters. This is achieved by *DNMT1* accessing hemi-methylated DNA during replication, priming the daughter unmethylated strand for methylation. However, anomalous DNMT-mediated methylation jeopardizes typical gene expression patterns as a result of increased or decreased accessibility of CpG-rich promoters. HCC and its adjacent tissues have demonstrated notably different DNA methylation patterns [6]. Where the noncancerous neighboring tissues display uniform and stable methylation patterns, HCC exhibits a marked heterogeneity. According to the reported results, HCC tissues manifest reduced methylation of CpG regions.  Table 1 shows a snippet of the reported signature of methylated genes in HCC, which is reportedly capable of differentiating HCC samples from neighboring tissues. A former study showed that DNA methylation of CpG island-associated promoters silenced gene expression and defined 222 drivers of epigenetic changes exhibiting this negative correlation. A preponderance of these candidate drivers was found to be enriched in inflammatory responses, a number of metabolic processes, and oxidation-reduction reactions. A set of reliable and robust candidates was also defined (Table 1).

*Neurofilament*, *heavy polypeptide* (*NEFH*) and *sphingomyelin phosphodiesterase 3* (*SMPD3*) were also defined as tumor suppressor genes that were hypermethylated and silenced in HCC [13]. The results obtained from the gain of function experiments revealed diminished cellular proliferation, whereas those of knockdowns restored tumor invasiveness and migratory capacities. Conversely, hypomethylation of the fetal promoters of the oncogene, *IGF2*, gave way to its overexpression, imparting virulent phenotypes [14]. DNA methylation has also been inculpated in the dysregulation of several long noncoding RNAs (lncRNAs), which have been awhile associated with HCC. The histone methyltransferase *enhancer of zeste homolog 2* (*EZH2*), which catalyzed the trimethylation at lysine 27 of histone H3, has been proven to silence *TCAM1P-004* and *RP11-598D14.1*: two tumor-suppressing long noncoding RNAs [15]. This has been supposed to be assisted by *Yin Yang 1* (*YY1*), which purportedly aids in recruiting *EZH2* to promoters of target genes [16]. The downregulation of these lncRNAs correlated with tumor progression owing to the inhibition of their moderation of the *mitogen-activated protein kinase* (*MAPK*), *tumor protein p53* (*p53*), and *hypoxia-inducible factor 1-alpha* (*HIF1-α*) pathways [15]. As would be expected, upregulation of histone methyltransferases might just be the driver for neoplastic events, given their downstream action on key promoters. By way of instance, *SET domain bifurcated histone lysine methyltransferase 1* (*SETDB1*), an H3K9-specific methyltransferase, has been reported to exhibit the most substantial increase in HCC in comparison to other epigenetic regulators [17]. *SETDB1* was shown to owe its overexpression in HCC to a gene duplication event, with an additional copy of chromosome 1q21 [17]. However, other anomalous events were discovered to contribute to its elevated levels, such as regulation by microRNAs (discussed below), or transcriptional activation such as this mediated by *specificity protein 1* (*SP1*) [17].

### 2.3. DNMT3

Contrary to *DNMT1*, *DNMT3a* and *DNMT3b* do not recognize hemimethylated DNA. They do not produce or maintain particular patterns of methylation [18], and they are not specifically associated with replication sites [19] as *DNMT1*. Rather, they mediate *de novo* methylation as mentioned previously. Additionally, it has been assumed that these DNMTs employ mechanisms different from *DNMT1* to access the heterochromatin [20], given the fact that they were found not to be associated with replication sites.

DNMT3 has been implicated in hepatocarcinogenesis. It has been expressly associated with hypermethylation of promoters controlling 22 tumor suppressor genes [21]. *DNMT3b* also exhibited a 4-fold increase of expression in HCC when compared to healthy livers, which correlated with poorer prognosis [21], which corroborates assumptions that *DNMT3* subtypes become overexpressed in cancer after having been downregulated postcellular differentiation [22].

In HCC of HBV etiology, the normally silenced *metastasis-associated protein 1* (*MTA1*) gene was upregulated by recruitment of *DNMT3a* and *DNMT3b* leading to hypomethylation of its promoter and increasing the tumor metastatic disposition [23]. Additionally, *DNMT3b* was elsewhere reported to be overexpressed by *telomerase reverse transcriptase* (*TERT*) in HCC. The resulting anomalous methylation patterns prompted activation of *AKT* [24]. Apart from its methylating capacity, *DNMT3b* was found to directly target *metastasis suppressor 1* (*MTSS1*), by direct binding to its promoter [25].

The implication of *DNMT3a* in HCC has also been corroborated. In a study by Zao et al., *DNMT3a* knockdowns displayed arrested cellular proliferation. Microarray analysis revealed concomitant upregulation of 153 genes, the preponderance of which bears CpG islands in their promoters. Among these activated genes was the tumor suppressor *PTEN* gene [26]. Moreover, *DNMTa* guided a conjectured distinction in the epigenetic dysregulation between different forms of liver cancer, where nonfibrolamellar HCC displayed significantly higher levels of *DNMTa* compared to the fibrolamellar variant [27]. This discrepancy was suggested to betray divergent epigenetic mechanisms in different HCC subtypes.

### 2.4. DNMT3L

Structurally similar and functionally complementary to *DNMT3a* and *DNMT3b* is *DNMT3L*, which, despite lacking intrinsic catalytic activity, enhances the binding of the former to S-adenosyl-L-methionine, the donor of the methyl group. Understanding the role of *DNMT3L* in full requires further analysis [28].

Given all of the above, it is clear that modifying any of these anomalies could potentially serve as a therapeutic modality in HCC. Below the major DNMT inhibitors with reported activity in HCC are outlined.

### 2.5. DNMT Inhibitors

Herein, the most prominent inhibitors of DNMT in HCC are outlined. Despite the fact that—in many instances—DNMT inhibitors may not be selective for one subtype over the other, the following is reported according to what the original account relayed. DNMT inhibitors are summarized in Table 2.

### 2.6. 5-Azacytidine

5-Azacytidine (5-AZA) is a synthetic analog of the nucleoside cytidine and an established inhibitor of *DNMT1*, marketed under the name Vidaza. In the context of HCC, treatment with 5-AZA conduced to tumor regression and a shift to a more differentiated phenotype, which was associated with regional demethylation of CpG regions upstream of the liver-specific genes *SLC10A1*, *CYP3A4*, *ALB*, and *miR-122*, which were downregulated pretreatments [29]. Additionally, this epigenetic modulation boosted the effects of sorafenib. 5-AZA triggered demethylation of 5-hydroxymethylcytosine (5hmC) via the *ten-eleven translocation proteins 2 and 3* [30]. *DNMT1* inhibition by 5-AZA was also found to synergize with immunotherapy via encouraging trafficking of T-cells to the tumor microenvironment secondary to a 5-AZA-induced upregulation of chemokine genes [31]. 5-AZA has been determined to be potentiated by sundry supplementation, such as vitamin C [32] and alendronate [33]. More recently, 5-aza-2′-deoxycytidine (5-Aza-CdR), a derivative of 5-AZA, was reported to downregulate *DNMT1*, *DNMT3a*, and *DNMT3b* [34].

### 2.7. Decitabine

Decitabine (5-aza-2′-deoxycytidine) is another analog of cytidine that also acts by blocking *DNMT1*. Decitabine was reported to demethylate the promoter of the *p16INK4A* gene, the product of which functions to regulate the cyclin-dependent kinases 4 and 6, leading to an upsurge of *p16INK4A* transcripts with ensuing G1 cell cycle arrest and a rise of the senescence-associated *β*-galactosidase [35]. Expression levels of *PRSS3* were also reported to rise in decitabine-treated cells [36]. The desilencing of *PRSS3* decelerated cellular proliferation due to inhibition of two cyclin/CDK complexes and downshifted migration through silencing *matrix metalloproteinase 2* (*MMP2*). A phase I/II clinical trial [37] scrutinized the efficacy of decitabine and its safety in advanced HCC. Western blots from patients' peripheral blood mononuclear cells (PBMCs) indicated decreased levels of DNMT1 in decitabine-treated participants.

### 2.8. Guadecitabine

Guadecitabine is a dinucleotide derivative of decitabine in which the latter is attached to a deoxyguanosine is by a phosphodiester bridge. Guadecitabine is commonly designated as SGI-110 and exhibits a more sustained systemic effect than its parent compound. Demethylation and activation of the tumor suppressor genes *DLEC1*, *RUNX3*, and *CDKN2A* were observed following SGI-110 treatment of Huh7 and HepG2 cells. Although its demethylating effects were compromised in the presence of the histone H2A variant, macroH2A1, SGI-110 was still capable of restricting tumor growth, unlike decitabine [38]. Potentiation of the cytotoxicity of the platinum-based antineoplastic oxaliplatin was reported when a pretreatment of SGI-110 was coadministered [39]. The mechanistic basis of such a sensitization involves counteracting the extensive methylation of targets within the *Wnt/EGF/IGF* signaling loop.

### 2.9. Zebularine

In HepG2 cells cultured at high densities, zebularine, a more stable and less toxic analog of 5-AZA [40], demonstrated a progressive escalation of expression of differentiation-associated genes and fomented apoptosis. shRNA-induced *DNMT1* knockdown annulled these effects [41]. Paradoxically, contrary reports indicated that zebularine had negligible influence on DNA methylation in the same cell line [42]. Despite the previous report, zebularine did affect several cytotoxic events, which have been attributed to mechanisms other than DNMT inhibition. Zebularine was found to inhibit *histone deacetylases* (*HDACs*) alongside DNMT genes in LS 174T cells [43]. *DNMT1*, *DNMT3a*, and *DNMT3a* as well as Class I HDACs and Class II HDACs were downregulated with a concomitant elevation in the expression of *p21Cip1/Waf1/Sdi1*, *p27Kip1*, and *p57Kip2* on treatment with zebularine, albeit to a more modest extent in comparison with trichostatin A. In the same study, it was observed that both agents acted synergistically to substantially increase apoptosis. It would thus seem propitious to examine these regulatory loops more closely in HCC.

### 2.10. Genistein

Genistein (GE) is an isoflavone derived from soybean and is characterized by its propensity to bind the estrogen receptor. GE upregulated cytochromes *1A1* and *1B1* in HT29 cells and downregulated cytochromes *26A1* and *26B1* [44]. In Hep3B cells, GE increased levels of phospho-*AMPK*, which mitigated inflammatory processes and consequent liver damage [45]. In concert with trichostatin A (TSA), GE restored the expression of the DNA methyltransferases *DNMT1*, *DNMT3a*, and *DNMT3b* in HepG2 cells [46]. GE exhibited biphasic effects at different concentration ranges, where at a low concentration of 1 *μ*M, it encouraged cellular growth, while at higher concentration within the range of 10-40 *μ*M, GE had antiproliferative effects. Proapoptotic effects were evident at all concentrations, unlike TSA, whose effects were observable only following a 3-day long treatment [47].

### 2.11. Epigallocatechin-3-Gallate (EGCG)

EGCG is the most abundant catechin in green tea that—among other flavonoids and catechins—has repeatedly been reported to possess tumor chemopreventive and antineoplastic effects in HCC [48]. EGCG has been shown to interact with the following amino acid residues within the catalytic domain of DNMT: P-1223, C-1225, S-1229, E-1265, and R-1309 [49, 50]. Moreover, catechol-containing polyphenols, of which EGCG is a member, inhibit DNMTs by mediating a rise in SAM O-methylation via catechol-O-methyltransferase. Alternatively, SAM levels were increased following disruption of the folate cycle secondary to dihydrofolate reductase inhibition by catechol-containing polyphenols. Direct inhibition of DNMTs by this class of compounds can also occur regardless of the methylation pattern [49, 50].

Additionally, EGCG has been shown to mediate a metabolic shift away from glycolysis in HCC cells, thereby promoting apoptosis and stunting cellular proliferation [51]. Mechanistically, this action has been attributed to its suppression of phosphofructokinase activity, whereby cellular stress is effected, ultimately culminating in programmed cell death. What is more, EGCG synergistically acted to ameliorate the antiproliferative effects of sorafenib [51]. Synergy between EGCG and metformin, the famous antidiabetic biguanide, has also been reported [52]. An EGCG/metformin combination therapy was associated with a significant reduction in *glypican-3*, *survivin*, *cyclin D1*, *VEGF*, and the long noncoding RNA *AF085935* and an elevation of the levels of *caspase 3* [52]. Another study examined the therapeutic effects of Y6, a chemically modified form of EGCG [53]. Again, and similar to its parent compound, Y6 efficiently curbed cellular proliferation. Additionally, it engendered a reversal of doxorubicin resistance in resistant BEL-7404 cells. The antiproliferative and antiapoptotic effects of Y6 correlated with reduced *P-glycoprotein 1* (*P-gp*) and *HIF1-α* on the mRNA and protein levels and was exacerbated in groups receiving Y6/doxorubicin combination therapy, compared to those on doxorubicin monotherapy. A compendium of studies reporting disease-modifying capabilities of EGCG in HCC can be found in a recent review by Bimonte et al. [48].

Other inhibitors of DNMT such as hydralazine, procainamide, and RG108 have been tested for their efficacy in cancer [11] but are yet to be examined as potential therapies in HCC.

## 3. Histone Modifications

**Figure 1:**
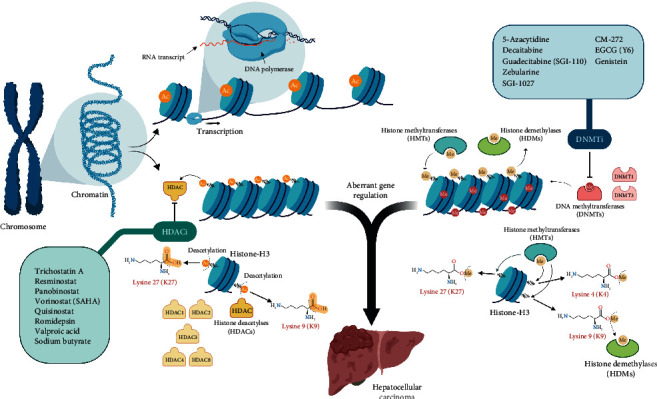
Epigenetic modulation of chromatin by histone deacetylation and methylation/demethylation as well as DNA methylation. The figure highlights the role of histone deacetylases (HDACs), histone methyltransferase (HMTs), histone demethylases (HDMs), and DNA methyltransferases (DNMTs) in creating the epigenetic signature observed in HCC in addition to their significance as targets for therapy. As is shown, the most common site for such modifications occurs on specific lysine residues on histone H3. “Created with BioRender.”

Chromatin is formed by the assembly of nucleosomal units, which are formed by the wounding of DNA around histone proteins. For accessing of genetic information, the highly packed chromatin has to be unwound. Chromatin modifications viz. methylation and acetylation are key controllers of this stipulation and thus play a crucial role in gene expression (Figure 1).

Histone modifications comprise sundry alterations to histone proteins including methylation (histone methyltransferases and histone demethylases), acetylation (histone acetyltransferases and histone deacetylases), ubiquitination, sumoylation, and phosphorylation [54]. The disruption of any of these modification patterns entails repercussions that may very well conduce to malignancy. However, for the purpose of this review, we elected to center this discourse on histone deacetylases (HDACs) given the abundance of data and the corroborated efficiency of HDAC inhibitors in preclinical and clinical settings [55]. Other reviews can be consulted for in-depth discussion of histone modifications and their implications in cancer [56–59].

Histone acetylation is controlled by two classes of enzymes: histone acetyltransferases (HATs) and histone deacetylases (HDACs). HATs catalyze the acetylation of lysine residues, whereas HDACs function to remove these acetyl groups [60].

As a result of acetylation, interaction between the histone octamers and DNA is compromised due to the neutralization of the positively charged lysine residues. The weakening of this interaction gives way to a transcriptionally permissive state of chromatin. HDACs promote an opposite effect, where the euchromatin state is favored as a consequence of retrieval of the positive charges on lysine residues, restoring the histone-DNA interaction [61]. A balance between HAT and HDAC activity ensures the maintenance of normal patterns of gene expression, and its disruption is often noted in many malignancies including HCC [62].

### 3.1. HDACs

There are around 18 HDACs, many of which have been shown to deacetylate nonhistone proteins [63]. Given the above, the centrality of HDACs to chromatin accessibility and control of gene expression [64] is obvious, and assumptions that HDACs constitute tumor suppressors or target for therapy are not only well-grounded but also experimentally evident.

In HCC, dysregulation of HDACs has been multiplied reported. By way of instance, *HDAC1* and *HDAC2* were found to be overexpressed in HCC patients of Southeast Asian origin and was associated with higher rates of mortality. Inhibition of these HDACs *in vitro* inhibited cellular proliferation [65]. The upregulation of *HDAC1* and *HDAC2* was found to suppress *fructose-1,6-bisphosphatase* (*FBP1*), a key enzyme in glycolysis [66], and *HDAC2* was further reported to modulate genes involved in the cell cycle and apoptosis [67]. *HDAC3* was recently demonstrated to be centrally implicated in hepatocarcinogenesis. Following a ubiquitination event, it dissociates from the *c-Myc* promoter, whereby K9 of histone H3 (H3K9) becomes acetylated and *c-Myc* is made transcriptionally available [68]. Elimination of *HDAC3* inhibited the trimethylation of H3K9 that occurs subsequent to the *HDAC3*-mediated deacetylation of this residue, arresting the contingent double-strand break repair mechanism and resulting in the accretion of bad DNA [69].

Interestingly, HDACs were also shown to counter cell migration. Acetylation of H3K4 and H3K56 within the *Snail2* promoter was markedly reduced in EMT thanks to *HDAC1* and *HDAC3* [70]. It is worthy to note that G9a, a histone H3 lysine 9 (H3K9) methyltransferase, has been recently recognized as vital for such *Snail2*-mediated inhibition of *E-cadherin* and consequent repression of mesenchymal properties [71]. It has even been targeted for therapy by administering its inhibitor, UNC0646, in nanodiamonds, which reduced H3K9 methylation and tumor invasiveness [72].

That being said, therapeutic inhibition of HDACs may sometimes prove problematic because of interference with various pathways [56] and, as evident above, for the bidirectional functionality it has sometimes demonstrated. It is thus of essence to dedicate some efforts to better understand and characterize the complex regulatory role of HDACs so as to determine their amenability to therapeutic targeting and define in what direction should therapeutic strategies be pursued.

### 3.2. HDAC Inhibitors

HDAC inhibitors (HDACi) are a group of agents that are useful in resolving aberrant patterns of deacetylation, modulating chromatin accessibility, the lack of which is often an inciting factor for tumorigenesis [73]. Below the most prominent HDACis are outlined (Table 3).

## 4. Hydroxamates

### 4.1. Trichostatin A

TSA is one of the most studied hydroxamate HDAC inhibitors. Following inhibition of HDACs 1, 2, and 3 by TSA, *apoptotic protease-activating factor 1* (*Apaf1*) was determined to become upregulated, which leads to the stimulation of mitochondrial caspase-driven apoptosis of the HLE and HLF HCC cell lines [74]. TSA was also found to restore the expression level of H2Aub, an H2A posttranslationally ubiquitinated at lysine 119, which is diminished in HCC. Simultaneously, TSA modulated the rates of H3S10 phosphorylation, which were inversely correlated with H2Aub in HCC [75]. In addition to *ubiquitin-specific peptidase 21* (*ups21*), which is responsible for the downregulation of H2Aub above, *CYLD* is another (lysine 63) deubiquitinase involved in the development of HCC. Contrary to *Ups21*, it is the inadequacy of *CYLD* that is associated with malignancy. TSA was shown to raise *CYLD* mRNA and protein levels in Huh7 and HepG2 cells [76]. Overexpression of ligands of *NKGD2* was noted following TSA treatment. It thus exerted its cytotoxic effect through stimulating natural killer (NK) cells to eliminate HCC cells [77]. Alternatively, the proapoptotic activity of TSA could be modulated by regulatory RNA species such as the long noncoding RNA, *lncRNA-uc002mbe.2*, which was increased post-TSA-treatment [78]. The proposed mechanism delineates an interaction between *lncRNA-uc002mbe.2* and *heterogeneous nuclear ribonucleoprotein A2B1* (*hnRNPA2B1*) which instigates the stimulation of *p21* and reduction of phosphorylated *AKT*. TSA has been used in conjunction with other agents such as sorafenib for enhancing therapeutic outcomes [79].

### 4.2. Resminostat

Resminostat is a pan-HDACi (inhibits both nuclear and cytoplasmic HDACs). In HepG2, SMMC-7721 and HepB3 cells, resminostat incited mitochondrial depolarization and apoptosis via the mitochondrial permeability transition pore pathway. It also evoked the production of *caspase 9* and *cytochrome c* [80]. The cytotoxic effects of resminostat were reinforced by inhibitors of the *mammalian target of rapamycin* (*mTOR*), which has been characterized as a resistance factor of resminostat [81]. The synergistic effects of resminostat with sorafenib have been repeatedly studied. The combination proved safe and effective. Resminostat shifted the cells from a mesenchymal to an epithelial phenotype, which better sensitized the cells to subsequent sorafenib treatment [82]. That being said, further investigation into the advantage of this combination is required. While an exploratory clinical study corroborates the above observations [83], another phase I/II study refuted an added utility of resminostat supplementation over sorafenib monotherapy [84].

### 4.3. Panobinostat (PANB)

Another potent pan-HDACi is PANB. Studies have shown that PANB affected a negative interference with DNMTs (as outlined in Table 2) and an ensuing impedance of methylation of classically hypermethylated genes, such as *APC* and *RASSF1A* [85]. PANB encouraged an increase of autophagic factors *Beclin1* and *Map1LC3B*, which concomitantly presented with the appearance of quasiautophagosome clusters along with the nuclear translocation of *p53* and *p73* in HepG2 and Hep3B cells, respectively, and regulation of *DRAM1* [86]. Ingeniously, ^18^F probes have been used as PET tracers to monitor angiogenic progression following PANB therapy, through imaging of integrin *αvβ3*. These PET scans revealed a substantially reduced uptake in HepG2 but not in HT29 neoplasm, in response to therapy in nude mice [87].

### 4.4. Vorinostat (VORN; SAHA)

Beyond chromatin unwounding, evidences have been provided that substantiate a role of VORN in initiating tumor hypoxia. Ostensibly, VORN-mediated acetylation of *heat shock protein 90* (*Hsp90*), a chaperone of *HIF-α*, hinders its nuclear translocation and forestalls its transcriptional activity [73]. As a result, levels of several downstream hypoxia-triggered molecules come to be deficient. VORN was used as an adjuvant to a number of anticancer drugs such as oxaliplatin [88] and the *mTOR* inhibitor, sirolimus [89]. Compared to 5-aza-2′-deoxycytidine (5-Aza-CdR), VORN exhibited superior apoptotic effects which was coincident with its inhibition of *HDAC1*. However, a combination of the two achieved maximal apoptosis of LCL-PI 11 cells [34].

### 4.5. Belinostat

Belinostat has been studied extensively but sporadically in different cancer types, mostly on hematologic malignancies. Despite its consistently promising results, belinostat remains underinvestigated in HCC. Hereunder, most of the reports on belinostat use in HCC are summarized. A multicenter phase I/II study aimed at determining the drug pharmacokinetic and toxicity profiles constitutes one major such report. The outcomes of the study were favorable in terms of disease stabilization (assessed via histoscores) and high tolerance to the drug, which is reflected in its outspread pharmaceutical window [78]. When combined with the checkpoint inhibitors anti-*PD-1* and anti-*CTLA-4* antibodies, belinostat potentiated the latter but not the former. The synergy was credited to a drop of regulatory T cells and a boosted *IFN-γ* production by T cells in the tumor microenvironment [90]. Withal, *PD-L1* inhibition was proposed, given its observed overexpression on antigen-presenting cancer cells and its retarded expression on effector T cells. Boron-incorporating prodrugs of belinostat have been propounded for improving its potency against solid tumors [91]. The prodrug form manifested superior bioavailability. However, the efficacy of this form remains to be examined in HCC.

## 5. Aliphatic Fatty Acids

### 5.1. Valproic Acid (VPA)

VPA, a class I and IIa HDACi, has a certain favorability to it, given its reasonable cost and wide safety margin. VPA demonstrated antineoplastic effects in PLC/PRF5 and HepG2 cells [92]. Moreover, VPA was shown to mediate a dissemination of its anticancer activity through its indirect modulation of cell-free DNA. This rather unique study was conducted under the hypothesis that cfDNA can mediate intercellular signaling. The cfDNA derived from VPA-treated cells induced glycolysis in naïve HepG2 cells. Subsequent analysis of the cfDNA from these cells revealed altered characteristics. As such, it was suggested that VPA treatment can be temporarily propagated across cells via their released cfDNA [93]. VPA rendered Hep3B cells more vulnerable to proton irradiation, protracting the actuated DNA damage, and promoted irradiation-mediated apoptosis [94]. Curiously, VPA increased irradiation-induced reactive oxygen species (ROS) production and silenced *nuclear factor erythroid 2-related factor 2* (*Nrf2*), which is quickly becoming a marker of radioresistance. VPA has been used in combination with doxorubicin [95] and sorafenib [96] and boosted the cytotoxic effects of cytokine-induced killer cells [97]. Recently, VPA was assessed alongside zebularine as to the effect on *Suppressor of cytokine signaling 1* (*SOCS-1*) and *Suppressor of cytokine signaling 3* (*SOCS-3*) expression [98]. Despite both suppressing cellular growth, only VPA demonstrated an apoptotic effect and correlated with an upregulation of *SOCS-1* and *SOCS-3*.

### 5.2. Sodium Butyrate

Butyrate is among the short chain fatty acids that are produced as a result of the anaerobic fermentation undergone by gut microbiota, and its benefits in restraining tumor growth have been documented. The sodium salt of butyrate has been explored as an epigenetic modulator in various malignancies. However, there remains a need for exploring its utility in HCC. Elevation of ROS and consequent autophagy were noted in Huh7 cells following butyrate treatment. Levels of phosphorylated *AKT* and *mTOR* were positively inhibited, which gave to a dependent rise in *ATG5*, *Beclin1*, and *LC3-II*, with subsequent assembly of the autophagosome machinery [99]. Otherwise, as noted with TSA (above), butyrate spurred on the expression of the deubiquitinase *CYLD* in Huh7 and HepG2 cells (Kotantaki & Mosialos, 2016).

## 6. Noncoding RNAs

### 6.1. MicroRNAs

**Figure 2:**
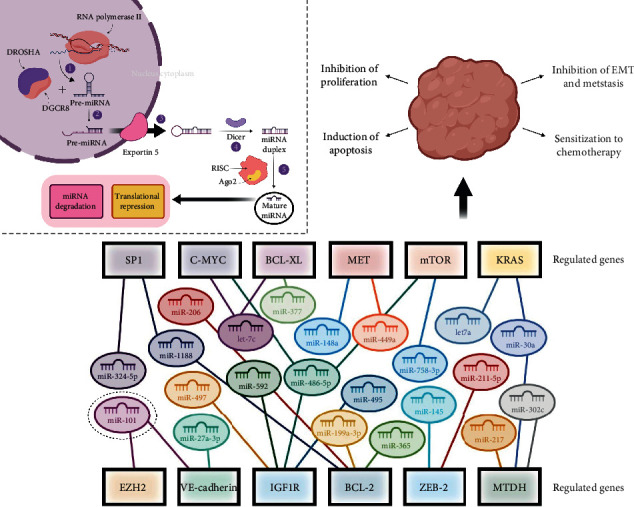
A schematic showing a network of several miRNAs with converging regulatory pathways in HCC therapy. The figure shows miRNAs sharing a common target as well as targets regulated by more than one miRNA. The therapeutic effects associated with all of the microRNAs in the illustrated panel correlate with their upregulation, except for miR-101 (marked). “Created with BioRender.”

MicroRNAs (miRNAs) are probably the most frequently studied biomolecules in cancer, and for a good reason. Given their integral role in gene expression manipulation, abnormal miRNome lies at the heart of the genetic dysregulation that predisposes to oncogenesis. miRNAs are encoded mostly in intergenic regions of the genome and are transcribed by RNA polymerase II. Following transcription, a primary RNA transcript forms a hairpin loop with terminal single-stranded extensions (Figure 2). Both the 5′ and 3′ extensions are cleaved off by a microprocessing complex made up of *DROSHA*, a class 2 RNase III and its accessory protein *DGCR8*, yielding what is referred to as a precursor miRNA (pre-miRNA) (Figure 2). The pre-miRNA is exported to the cytoplasm shuttled through nuclear pores by the transporter *exportin 5* (Figure 2). In the cytoplasm, the pre-miRNA is recognized by the *TRPB2*-bound enzyme *Dicer*, another RNase III, which clips off the loop, producing a double-stranded miRNA (ds-miRNA or miR/miR∗ duplex) (Figure 2). The Argonaut protein, *Ago2*, interacts with *Dicer* to bind the ds-miRNA, unwinding the miRNA duplex, releasing the passenger strand that is degraded and retains the guide strand (Figure 2), which is 15-25 nucleotides long [100, 101]. Along with *Ago2*, the guide strand interacts with a group of proteins forming the *RNA-induced silencing complex* (*RISC*) which constitutes the active silencing species. Complementarity with the 3′UTR of target mRNAs determines which are marked for silencing, which is further reinforced by near-perfect complementarity of the mRNA with the miRNA seed sequence. The bound mRNA may be degraded or its translation impeded, turning off the mRNA-encoding gene. Hereinafter, some of the most therapeutically bioactive miRNAs are explored.

### 6.2. miR-126

*miR-126* was shown to target *EGFL7* and *VEGF* in HCC tissues, lowering their expression [102]. Gain of function studies demonstrated that this regulatory mechanism resulted in significant reduction of tumor size and weight as well as a decreased microvascular density of transplanted neoplasms. Other studies further corroborated the antiangiogenic role of *miR-126*. *miR-126*-transfected HepG2 cells were transplanted in nude mice in parallel with a control group receiving a transplant of nontransfected cells. Postresection analysis revealed lower VEGF expression levels in the *miR-126* group compared with controls as well as relatively reduced tumor volumes [103]. Du and colleagues [104] reported similar findings for the 3p arm of *miR-126*. According to the results of their experiments, *miR-126-3p* gain of function inhibited expansion of tumor vasculature and reduced microvascular density and capillary tube formation. *Low-density lipoprotein receptor-related protein 6* (*LRP6*) and *phosphoinositide-3-kinase regulatory subunit 2* (*PIK3R2*) were identified as the direct targets, and their silencing occasioned similar effects to those brought about by overexpression of *miR-126-3p*. Beyond its effects on tumor vascularization, *miR-126* has manifested antiproliferative and antiapoptotic functionalities. Zhao et al. [105] reported *sex-determining region Y-box 2* (*SOX2*) as a putative target of *miR-126*. *miR-126* mimics correlated with downregulated levels of *SOX2* and subsequent cell cycle arrest and apoptosis in HepG2 cells. In addition to the above, *miR-126* repressed metastatic capability of HCC. A negative correlation between *miR-126* and *ADAM metallopeptidase domain 9* (*ADAM9*) has been established in hepatitis B virus-related HCC [106]. Upregulation of *miR-126* attenuated *ADAM9* expression and consequently inhibited tumor migration and reduced instances of metastases. Ectopic expression of *miR-126* was associated with failure of *miR-126*-trasnfected SMMC-7721 cells to achieve pulmonary colonization *in vivo* [107]. The *miR-126-3p/PIK3R2/LRP6* regulatory loop mentioned above has also been proven to result in the suppression of cellular migration, ECM invasion, and tumor metastasis [104].

### 6.3. miR-148a

*miR-148a* has recently been shown to posttranscriptionally regulate the expression of *transferrin receptor 1* (*TFR1*) [108]. Given the negative correlation observed, an increase in *miR-148a* levels is surmised to downregulate *TLR1* in HCC, resulting in reduced uptake of transferrin-bound iron by the cancer cells, which consequently leads to a drop in cellular iron levels, suppressing proliferation. The closely related *miR-148b* is purported to directly target *Rho-associated protein kinase 1* (*ROCK1*) to similar antiproliferative effects [109]. Other endeavors indicated that *miR-148a* mimics might be implicated in the regulation of hepatocytic differentiation via regulating the *IKKα/NUMB/NOTCH* pathway [110]. Furthermore, *miR-148a* positively correlated with the expression of *E-cadherin* and downregulated mesenchymal markers, i.e., *vimentin*, *fibronectin*, and *N-cadherin* in hepatoma cells, by binding and inhibiting *Met* and attenuating its downstream signaling, ultimately resulting in decreased nuclear accumulation of *SNAIL* [111]. As such, *miR-148a* was effective in discouraging EMT and suppressing pulmonary metastasis. A number of studies sought to examine the role of microRNAs in regulating hepatic stellate cells (HSCs), to outstanding outcomes. *miR-148a* was shown to target and inhibit *growth arrest-specific gene 1* (*Gas1*) mRNAs, thwarting Hedgehog signaling and preventing biogenesis of autophagosomes, which manifested as enhanced autophagy and apoptosis of HSCs [112]. Interestingly, *miR-148a* itself has been shown to be epigenetically regulated in HCC. By virtue of its hypermethylated CpG island, *miR-148a* is typically silenced in HCC cell lines [113]. Ironically, *DNMT1*, an established target of *miR-148a*, is the DNA methyltransferase that mediates such hypermethylation. *DNMT1* is upregulated in HCC, and thus, it downplays its primary regulator by a negative feedback loop. Fortunately, ectopic expression of *miR-148a* abrogates the inhibitory effects of *DNMT1*, permitting its regulatory role to take effect.

### 6.4. miR-199a

*miR-199a-3p* prompted a diminution of malignant nodular size and numbers in a transgenic mouse model that is prone to developing HCC, coinciding with a downregulation of its putative targets: *p21 activated kinase 4* (*PAK4*) and *mTOR*, and hence a drop in the levels of *FOXM1*, replicating effects observed following treatment with sorafenib [114]. Targeted delivery of *miR-199a-3p* to neoplasms in nude mice displayed similar auspicious outcomes. Mimics of the 3p arm of *miR-199a* were encapsulated in bionic acid- (BA-) functionalized peptide-based nanoparticles (NPs). Hepatospecific delivery was achieved through the high affinity interaction between BA and the asialoglycoprotein receptors, which are overly expressed in HCC cells. Mirroring *mTOR* inhibition *in vitro*, apoptotic and antiproliferative events were noted, following IV administration of the NPs [115]. Preceding *in vitro* analysis had additionally exposed an upregulation of *PUMA* secondary to a rise in *ZHX1* levels, concurring with repressed growth. Increased cell death was paralleled by *Bcl2* tapering off and accretion of *cleaved caspase 3* and *Bax* [116]. Both arms of *miR-199a* positively modulated *E-cadherin* through inhibition of its *Notch1*-mediated suppression [117], which also suggests a role for *miR-199a* in checking EMT. *miR-199a-5p* was also shown to restrain metastatic disposition by silencing *Snail1* [118]. The biotherapeutic activity of the 5p arm extends well beyond its regulation of *E-cadherin*. Upwards of EMT, introducing *miR-199a-5p* stifled *clathrin heavy chain* (*CTLC*) expression arresting cellular growth *in vitro* and xenograft mice models [119]. Moreover, *VEGF*-initiated cell proliferation was reportedly halted posttreatment with *miR-199a-5p*, thanks to its modulation of the *nitroreductase*, *NOR1* [120].

### 6.5. miR-503

Several studies reported antimetastatic effects of *miR-503* through dampening the expression of various targets such as *WEE1* [121], *PRMT1* [122], and *ARHGEF19* [123]. Decelerated cellular growth, inducement of apoptosis, and sensitization to chemotherapy were all events associated with *miR-503* gain of function and were collateral to its modulation of its determined targets viz. *eukaryotic translation initiation factor 4E* (*EIF4E*) [124] and *insulin-like growth factor 1 receptor* (*IGF-1R*) [125].

### 6.6. miR-101

*miR-101* has been a confirmed tumor suppressor and recurrently reported as a downregulated species in HCC. Marked clampdown of tumor growth has been linked to the modulation of the *HGF/c-MET* axis by *miR-101-3p* [126]. *miR-101* also attenuated the expression of the *zinc-finger protein 217* (*ZNF217*), a potent effector of malignant immortalization [127]. Further, vasculogenic mimicry, an insidious mechanism of *de novo* vasculogenesis by which cancer resists angiogenic arrest, was undermined by *miR-101* mimics, which sabotaged *TGF-β* and *SDF1* signaling in cancer-associated fibroblasts and impaired *VE-cadherin* expression [128]. Similar to *miR-503*, *miR-101-3p* also targeted *WEE1*, which was shown to sensitize Huh7 and PLC5 to radiotherapy, an effect that is partially abrogated in HCC by the lncRNA *nuclear-enriched abundant transcripts 1* and *2* (*NEAT1* and *NEAT2*) [129]. On top of that, *miR-101* subverted the *TGF-β1*-instigated build-up of extracellular matrix (ECM), reversing hepatic fibrosis, and blunted the levels of phosphorylated *PI3K*, *mTOR*, and *Akt* [130]. As with other epigenetic modulators, *miR-101* has been tried as a part of several combinatorial regimens. Synergy was reported with liposomal doxorubicin [131] and the lncRNA *LINC00052*, which promoted the expression of the 3p arm of *miR-101* that restricted the expression of *SRY-related HMG-box gene 9* (*SOX9*) [132].

As is evident in Figure 2 and Table 4, different miRNAs have common targets and inevitably a single target can be regulated by more than one miRNA, which creates an elaborate regulatory network and sometimes complicate the utilization of miRNAs for diagnostic and therapeutic purposes.

### 6.7. Long Noncoding RNAs

Another major class of nonprotein-coding RNAs that is central to HCC and which is gaining significant attention as of late is long noncoding RNAs (lncRNAs). lncRNAs are a bit longer than miRNAs with a transcript length of more than 200 nucleotides [133]. lncRNAs have been extensively researched for their role in HCC pathogenesis and their therapeutic potential. As will be exposited shortly, a number of lncRNAs function by what is known as miRNA sponges, which basically involves buffering the action of miRNAs on their target mRNAs.

Given the comprehensive nature of this review, only some of the most recent reports involving lncRNA in HCC are discussed below. However, detailed information about earlier reports can be found in the following reviews: [134–136]. Additionally, the following bibliographic data [134–214] afford an extensive exposition of the most recent HCC lncRNA-oriented work. Beside the compendious run-through below, Table 5 affords an encyclopedic overview of the lncRNAs studied in these resources which were not discussed in the text for practical reasons.

### 6.8. GAS8-AS1

It was recently reported that both the *GAS8* gene and its resident lncRNA, *GAS8-AS1*, act as tumor suppressors and manifest a significantly low expression in HCC tissues, which correlated with poor prognosis [157]. *GAS8-AS1* was curiously found to mediate the transcription of *GAS8*. It was essential in maintaining chromatin in an uncondensed state by recruiting the H3K4 methyltransferase *MLL1* and its accessory protein *WD-40 repeat protein 5* (*WDR5*). This leads to the potentiation of RNA polymerase II and enhanced transcription of *GAS8*. The above molecular events suppressed oncogenesis and impeded HCC development.

### 6.9. FENDRR

*FOXF1 adjacent noncoding developmental regulatory RNA* (*FENDRR*), another lncRNA that was found to be downregulated in HCC, was recently advocated as a potential therapeutic approach to arrest HCC progression and discourage metastasis. Ectopic expression of *FENDRR* was reported to check malignant growths *in vitro* and *in vivo*, as well as repressing HCC migration and invasion. This was purported to occur via epigenetic regulation of *glypican-3* (*GPC3*). Through interacting with the *GPC3* promoter and subsequently leading to its methylation, *FENDRR* functions to silence *GPC3*, counteracting the latter's oncogenic effects [168].

### 6.10. CASC2c

*Cancer susceptibility candidate 2c* (*CASC2c*) is one of three lncRNA transcripts produced by the alternative splicing of *cancer susceptibility 2* (*CASC2*). Inherently silenced in HCC, the overexpression of *CASC2c* resulted in the suppression of proliferation of HCC cells, while inducing apoptosis. These effects coincided with lowered phosphorylated *extracellular signal-regulated kinase 1/2* (*p-ERK1/2*) and *β-catenin* levels [201].

### 6.11. miR503HG

*miR503HG*, the host gene of *miR-503* (see above), has been found to be significantly downregulated in HCC [141]. This silencing was closely related to survival rates and duration until tumor recurrence and is thus conjectured to be a prognostic biomarker. The gain of function abrogated the invasion and metastasis of HCC cells. *miR503HG* was also found to promote the degradation of the *heterogeneous nuclear ribonucleoprotein A2/B1* (*HNRNPA2B1*) by ubiquitination and subsequent proteasomal degradation, which consequently led to the destabilization of *p52* and *p65* transcripts and ultimately suppressed *NF-κB* signaling in HCC. Given their innate interplay and their common effect on HCC cells, *miR503HG* and its resident microRNA (*miR-503*) could cooperatively function to stymie migration of HCC cells.

### 6.12. LINC00467

*LINC00467*, another lncRNA that was found to be downregulated in HCC, has been studied as a potential therapeutic target thanks to its role as an antagomir for *miR-9-5a*, which targets *peroxisome proliferator-activated receptor alpha* (*PPARA*) for silencing [140]. *LINC00467* ectopically expressed in HCC cells conduced to antiproliferative effects and, like *miR503HG*, checked migration and invasion. The authors propose a pivotal implication of the *LINC00467/miR-9-5p/PPARA* loop in the initiation and progression of HCC.

### 6.13. Linc-GALH and UC001kfo

Contrary to the above-mentioned lncRNAs, which are downregulated in HCC and which are considered tumor suppressors, other lncRNAs are oncogenic, with anomalously high expression in HCC. *Linc-GALH* and *UC001kfo* were recently reported to be upregulated in HCC. *Linc-GALH* was surmised to regulate methylation of *Gankyrin* and hence its expression [190]. Mechanistically, this was proposed to occur via deubiquitinating DNMT1. This promoted migration and invasion in HCC cells and was rescinded in silencing experiments. Increased expression of *UC001kfo* correlated with tumoral macrovascular invasion (MVI) and TNM staging of HCC, with higher levels predisposing to poorer prognoses [179]. *UC001fko* boosted tumor proliferation and EMT, presumably through targeting *alpha-smooth muscle actin* (*α-SMA*). The authors indicate the potential of *UC001kfo* to serve as a prognostic marker as well as a target for therapy.

### 6.14. LINC00346

*LINC00346* was shown to be aberrantly upregulated in HCC [139]. *LINC00346* enhanced the expression of *WD Repeat Domain 18* (*WDR18*) by virtue of competitively binding to *miR-542-3p*, a downregulated tumor suppressor in HCC cells. This sponging effect leads to the activation of the *Wnt/β-catenin* pathway. As such, *LINC00346* could be a viable target in HCC therapy, where its inhibition is presumed to unmask the anticancer effects of *miR-542-p*.

### 6.15. LINC00978

Both tumor tissues and serum samples from HCC patients manifested an exaggerated expression of *LINC00978* [69]. Serum levels of this lncRNA could even distinguish between HCC patients and patients with hepatitis or cirrhosis. *LINC00978* was reported to promote cellular proliferation, migration, and invasion, wherein its knockdown arrested the cell cycle and encouraged apoptosis. The authors unveiled the mechanistic basis of such effects to involve binding of *LINC00978* to *EZH2*, leading to its buildup at the promoter regions of *E-cadherin* and *p21* genes, which leads to these genes becoming silenced subsequent of *EZH2*-mediated H27K3 trimethylation. The validity of this regulatory circuit was confirmed by the abrogation of *LINC00978* knockdown's inhibitory effects in *E-cadherin* and *p21* knockdowns.

### 6.16. NEAT1

*Nuclear-enriched abundant transcript 1* (*NEAT1*) is another lncRNA that is upregulated in HCC [138]. Silencing of *NEAT1* compromised cell viability and was shown to be proapoptotic in HepG2 and Huh7 cells. Again, as with other lncRNA/miRNA-negative correlations, *NEAT1* exhibited an opposite trend of expression to *miR-129-5p* in HCC. Ectopic expression of *NEAT1* suppressed *miR-129-5p* via modulating the *valosin-containing protein* (*VCP*)*/IκB* axis to the overall result of encouraging cellular proliferation.

### 6.17. ANRIL, LINC01296, and LINC01224

Similarly, *antisense noncoding RNA in the INK4 locus* (*ANRIL*), *LINC01296*, and *LINC01224* were all overexpressed in HCC and mediated their oncogenic effects through inhibition of microRNA signaling axes. *ANRIL*'s prooncogenic effects were found to rely on its suppression of *miR-384*, which targets *signal transducer and activator of transcription 3* (*STAT3*) [214]. These correlations were observed both *in vitro* and *in vivo*. *LINC01296* regulated the *miR-26a/PTEN* axis, resulting in tumor progression also *in vitro* and *in vivo* [137]. Similarly, an upswing of *LINC01224* in HCC was correlated with a silenced *miR-330-5p* and a consequent upregulation of its target, *checkpoint kinase 1* (*CHEK1*) [212]. *LINC01224* knockdowns exhibited a concurrent downregulation of *CHEK1*, owing to its binding to and inhibition of *miR-330-5p*, leading to tumor regression.

### 6.18. ZFAS1

HCC tissues exhibited an increased level of *ZFAS1*, compared to neighboring normal tissues [69]. The proliferative capacity of the tumor was substantially compromised subsequent of *ZFAS1* silencing, and its overexpression had a gainful effect on tumor growth. The authors report that the tumor suppressor miRNA, *miR-193a-3p*, was elevated in *ZFAS1* knockdowns which, confirmed by luciferase reporter assay and correlation analysis, suggested that the prooncogenic role of *ZFAS1* relied on the suppression of *miR-193a-3p*.

### 6.19. CRNDE

The *colorectal neoplasia differentially expressed* (*CRNDE*) lncRNA has recently been proven to be yet another prooncogenic lncRNA in HCC [210]. Its overexpression was associated with an enhanced proliferative and migratory competence of HCC cells, not to mention an ameliorated resistance to chemotherapy. *CRNDE* was determined to inhibit the Hippo pathway and encourage the *EZH2*-, *SUV39H1*-, and *SUZ12*-mediated inhibition of tumor suppressor genes viz. *large tumor suppressor 2* (*LATS2*) and *CUGBP Elav-like family member 2* (*CELF2*).

### 6.20. MALAT1

MALAT1 is a notoriously tumorigenic lncRNA implicated in many cancers. Recently, Chang et al. [209] proposed exploiting a *MALAT1/Wnt* regulatory loop for therapeutic purposes in HCC. They reported that *MALAT1* knockdowns evidenced a suppression of canonical *Wnt* signaling and impaired tumorsphere formation, which was coincident with a decline in CD90+ and CD133+ cells, which consolidated the hypothesis that *MALAT1* plays a vital role in promoting stemness in HCC cells.

## 7. Future Perspective

Despite the thorough study of epigenetic modulators, their extension to the clinical setting stands far from realizable. Further research mindful of the efficacy versus long-term toxicity/of these alternative strategies should be advocated. Studies looking into the pharmacokinetics of these agents as well as others seeking efficient targeted delivery with minimal systemic side effects are warranted. Addressing the adaptability of these modes of treatment to the clinic can bring us a long way, especially with the dosing curtailment of the highly toxic agents afforded by the concomitant use of the suggested alternatives, which, in some instances, may completely replace current debilitating treatments. As was mentioned, various exploratory clinical studies were carried out, but these need to be seen through to subsequent trial phases and on larger populations. Fortunately, the possible risk posed by a preponderance of these modulators is not significant to impede but should embolden such undertakings.

In addition to the clinical application, endeavors oriented to further our understanding of the elaborate epigenome and its regulation remain imperative. New epigenetic mechanisms are still being discovered contemporarily and progress in the field could do with pursuing modulators of these and assessing their benefits over the already defined ones. For example, decreased crotonylation of histone lysines has been recently incriminated in the progression of HCC [215]. This discovery should prompt several spin-offs in which the enhancers of crotonylation are suggested and assessed for therapeutic utility. Several defined modulatory agents such as histone demethylases (specifically Jumonji lysine demethylases) and helicases (HELLS) [216] among others also remain underresearched in HCC and should thus constitute a future research direction in HCC therapeutics.

## 8. Conclusion

The modulation of the altered epigenome in HCC is a promising therapeutic strategy. Verified potency and tenability to formulation demands for maximal systemic effects render many of the hereinabove nominated agents an intriguing recourse that could be subsequently implemented in clinical settings as a standalone curative or a potentiating adjuvant. It would also remain of equal importance to examine if these modulators can act in parallel to attenuate metastasis. More importantly, validating the use of these modulators in the treatment of HCC with different etiologies will aid in paving the road for personalized medicine together with the advancements in the pharmacogenomics/pharmacogenetics field. This holistic approach is forecasted to lower the success barrier, at least in part, in the treatment of HCC.

## Figures and Tables

**Table 1:** Aberrant methylation patterns in hepatocellular carcinoma (HCC). A comprehensive list of genes, which were dysregulated in HCC due to aberrant methylation patterns.

Gene	Methylation pattern	Ref.
*ACSL4*	Hypomethylation	[217]
*ALDH3A1*	Hypomethylation	
*APOA5*	Hypermethylation	
*CLDN15*	Hypomethylation	
*CDKN2A*	Hypermethylation	[6]
*CYP7A1*	Hypomethylation	[217]
*DEFB119*	Hypomethylation	[6]
*DPP6*	Hypomethylation	
*ENDOD1*	Hypermethylation	[217]
*EZR*	Hypermethylation	
*GLUL*	Hypomethylation	
*GZMB*	Hypomethylation	[6]
*MIR21*	Hypomethylation	[218]
*Myo1g*	Hypermethylation	[219]
*NEFH*	Hypermethylation	[13]
*NKX3-2*	Hypermethylation	[6]
*NDRG2*	Hypermethylation	
*PDE1A*	Hypomethylation	
*PHYHD1*	Hypermethylation	[217]
*PRH2*	Hypermethylation	[6]
*RASSF1A*	Hypermethylation	[220]
*RP11-598D14.1*	Hypermethylation	[15]
*SCAND3*	Hypermethylation	[219]
*SPP1*	Hypomethylation	[217]
*SPRR2A*	Hypomethylation	[6]
*SLC25A47*	Hypermethylation	[6]
*SLC25A47*	Hypermethylation	[217]
*SLC39A12*	Hypomethylation	[6]
*SMPD3*	Hypermethylation	[13]
*SFN*	Hypomethylation	[217]
*SGCA*	Hypomethylation	[6]
*TBX4*	Hypermethylation	
*TCAM1P-004*	Hypermethylation	[15]
*TKT*	Hypomethylation	[217]
*VTRNA2-1*	Hypermethylation	[221]
*ZPBP*	Hypermethylation	[6]

*ACSL4*: Acyl-CoA Synthetase Long Chain Family Member 4; *ALDH3A1*: Aldehyde Dehydrogenase 3 Family Member A1; *APOA5*: Apolipoprotein A5; CLDN15: Claudin-15; *CDKN2A*: cyclin-dependent kinase inhibitor 2A; *CYP7A1*: Cytochrome P450 Family 7 Subfamily A Member 1; *DEFB119*: Defensin *β* 119; *DPP6*: Dipeptidyl peptidase 6; *ENDOD1*: Endonuclease Domain Containing 1; *EZR*: Ezrin; *GLUL*: Glutamate-Ammonia Ligase; *GZMB*: Granzyme B; *MIR21*: microRNA-21; *Myo1g*: Myosin 1g; *NDRG2*: N-myc downstream-regulated gene family member 2; *NEFH*: Neurofilament, heavy polypeptide; *NKX3-2*: NK3 Homeobox 2; *PDE1A*: Phosphodiesterase 1A; *PHYHD1*: Phytanoyl-CoA Dioxygenase Domain Containing 1; *PRH2*: Proline-rich protein HaeIII subfamily 2; *RASSF1A:* Ras association domain family 1 isoform A; SCAND3: SCAN domain containing 3; *SFN*: Stratifin; *SGCA*: *α*-sarcoglycan; *SLC25A47*: Solute Carrier Family 25 Member 47; *SLC39A12*: Solute carrier family 39 member 12; *SMPD3*: sphingomyelin phosphodiesterase 3; *SPP1*: Secreted Phosphoprotein 1; *SPRR2A*: Small proline-rich protein 2A; *TBX4*: T-box 4; *TKT*: Transketolase; *VTRNA2-1*: Vault RNA 2–1; *ZPBP*: Zona pellucida binding protein.

**Table 2:** DNA methyltransferase (DNMT) inhibitors in HCC. The table shows the most prominent DNMT inhibitors, the changes in the targets of the inhibited DNMTs, and the resulting effects on the tumor.

DNMT inhibitor	DNMT targets affected	Effect	Ref.
5-Azacytidine	*SLC10A1*, *CYP3A4*, *ALB*, and *miR-122*	Inhibits tumor growth	[29]
Decaitabine	*p16INK4A* (activation)	G1 cell cycle arrest	[35]
	*PRSS3* (activation)	Inhibits proliferation and migration	[36]
Guadecitabine (SGI-110)	*DLEC1*, *RUNX3*, and *p16INK4A*	Inhibits tumor growth	[38]
Zebularine	*CDK2*, *Bcl-2*, and phosphorylation of *Rb* (inhibition) and *p21WAF/CIP1* and *p53* (activation)	Inhibits proliferation and induces apoptosis	[42]
SGI-1027	*Bcl-2* (inhibition) and *BAX* (activation)	Induces apoptosis	[222]
CM-272	*E-cadherin*, *CYP7A1*, *FBP1*, *GNMT*, and *MAT1A* (activation)	Inhibits proliferation and decreases adaptation to hypoxia	[223]
EGCG (Y6)	*P-gp* and *HIF1-α* (inhibition)	Inhibits proliferation and reverses doxorubicin-resistance	[53]
Genistein	*CYP1A1*, *CYP1B1*, and *p-AMPK* (activation) and *CYP26A1* and *CYP26B1* (inhibition)	Inhibits proliferation (at a 10-40 *μ*M concentration) and induces apoptosis	[44]

*ALB*: albumin; *BAX*: Bcl-2-like protein 4; *Bcl-2*: B-cell lymphoma 2; *CDK2*: cyclin-dependent kinase 2; *CYP1A1*: cytochrome P450 1A1; *CYP1B1*: cytochrome P450 1B1; *CYP26A1*: cytochrome P450 26A1; *CYP26B1*: cytochrome P450 26B1; *CYP3A4*: cytochrome P450 3A4; *CYP7A1*: cholesterol 7*α*-hydroxylase-1; *DLEC1*: deleted in lung and esophageal cancer 1; *FBP1*: fructose-1,6-bisphosphatase; *GNMT*: glycine-N-methyl transferase; *HIF1-α*: hypoxia-inducible factor 1-*α*; *MAT1A*: methionine-adenosyltransferase 1A; *p16INK4A*: cyclin-dependent kinase inhibitor 2A; *p21WAF/CIP1*: cyclin-dependent kinase inhibitor 1; *p53*: tumor protein p53; *p-AMPK*: phosphorylated AMP-activated protein kinase; *P-gp*: P-glycoprotein 1; *Rb*: retinoblastoma; *RUNX3*: RUNX Family Transcription Factor 3; *SLC10A1*: sodium/bile acid cotransporter.

**Table 3:** Histone deacetylase (HDAC) inhibitors in HCC. The table shows the most prominent HDAC inhibitors that have been studied in HCC, their cellular targets, and their antitumor effects.

Hydroxamates			
HDACi	Target(s)	Effect	Ref.
Trichostatin A	*Apaf1* and *H2Aub* (activation)	Promotes apoptosis	[74]
	*ULBP1/2/3* and *MICA/B* (Activation)	Inhibits tumor cell growth	[77]
Resminostat	*Caspase 9* and *cytochrome c* (activation)	Promotes mitochondrial depolarization and apoptosis	[80]
Panobinostat	*Beclin1*, *Map1LC3B*, and *p53* (activation) and *p73* nuclear translocation	Promotes autophagy	[86]
Vorinostat (SAHA)	*HIF-α* (inhibition)	Initiating tumor hypoxia	[73]
	*DR5* (activation) and *c-Flip* (inhibition)	Sensitization to TRAIL-induced apoptosis	[224]
Quisinostat (±sorafenib)	*c-Caspase 3*, *c-Caspase 9*, *c-PARP*, and *Bax* (activation) and *Bcl-xL*, *Bcl-2*, *survivin*, *PI3K-p110*, *PI3K-p85*, and *p-AKT* (inhibition)	Inducing G0/G1 phase arrest and apoptosis	[225]
Cyclic peptides			
Romidepsin	*p-Erk* and *p-JNK* (activation)	Induces cell cycle arrest in the G2/M phase and apoptosis	[226]
Aliphatic fatty acids			
Valproic acid	*Nrf2* (inhibition)	Sensitization to proton irradiation	[94]
Valproic acid (+DOX)	*AKT/mTOR* (inhibition)	Increases ROS and induces autophagy	[95]
Sodium butyrate	*p-AKT* and *mTOR* (inhibition) and *CYLD* (activation)	Increases ROS and induces autophagy	[99], [76]

*Bax*: Bcl-2-associated X protein; *Bcl-2*: B-cell lymphoma 2; *Bcl-xL*: B-cell lymphoma extra large; *c-Caspase 3*: cleaved caspase 3; *c-Caspase 9*: cleaved caspase 9; *c-PARP*: cleaved Poly (ADP-ribose) polymerase; *CYLD*: CYLD lysine 63 deubiquitinase; *DOX*: doxorubicin; *DR5*: death receptor 5; *mTOR*: mammalian target of rapamycin; *Nrf2*: nuclear factor erythroid 2-related factor 2; *p-AKT*: phosphorylated protein kinase B; *p-Erk*: phosphorylated extracellular-signal-regulated kinase; *PI3K-p110*: phosphatidylinositol 3-kinase subunit p110; *PI3K-p85*: phosphatidylinositol 3-kinase subunit p85; *p-JNK*: phosphorylated c-Jun N-terminal kinase; *ROS*: reactive oxygen species.

**Table 4:** MicroRNAs (miRNAs) with disease-modifying effects in HCC. The table shows the direction of microRNA expression associated with the therapeutic effects, the regulated targets, and the observed effects in HCC.

MicroRNA	Expression changes associated with therapeutic effects	Effect	Targets (and the direction of their therapeutic regulation)	Reference
*let-7c*	Upregulation	Induction of apoptosis and inhibition of proliferation	*LIN28B*, *ARID3B*, *Bcl-xL*, and *c-Myc* (downregulation)	[227]
*miR-663b*	Upregulation	Suppression of tumor proliferation and invasiveness	*GAB2* (downregulation)	[228]
*miR26a*	Upregulation	Growth inhibition, migration, invasion, colony formation; initiation of hepatoselective apoptosis. Enhancement of chemosensitivity	*CCND2*, *IL-6*, and *PIK3C2α* (downregulation)	[229, 230]
*miR-122*			*ADAM17*, *CCNG1*, *ADAM10*, and *Bcl-w* (downregulation)	
*miR-621*	Upregulation	Amelioration of tumor radiosensitivity	*SETDB1* (downregulation)	[231]
*miR-299-5p*	Downregulation	Suppression of proliferation, migration, and invasion; initiation of apoptosis	*SIAH1* (upregulation)	[232]
*miR-577*	Upregulation	Inhibition of EMT and metastasis	*HOXA1* (downregulation)	[233]
*miR-501-3p*	Upregulation	Inhibition of proliferation, EMT, migration, and invasion	*LIN7A* (Downregulation)	[234]
*miR-378a*	Upregulation	Inhibition of proliferation and enhancement of sensitivity to sorafenib-based chemotherapies	*VEGFR* , *PDGFRβ*, *MMP-2*, and *c-Raf* (downregulation)	[235]
*miR-204-5p*	Upregulation	Inhibition of cellular proliferation and clonogenicity	SIX1 (downregulation)	[236]
*miR-495*	Upregulation	Inhibition of proliferation and invasion	*IGF1R* (downregulation)	[237]
*miR-758-3p*	Upregulation	Inhibition of proliferation, migration, and invasion	*MDM2* and *mTOR* (downregulation)	[238]
*miR-30a-5p*	Upregulation	Inhibition of proliferation and invasion	*FOXA1* (downregulation)	[239]
*miR-196a*	Downregulation	Induction of apoptosis	*FOXO1* (upregulation)	[240]
*miR-30a*	Upregulation	Induction of apoptosis	*KRAS* (downregulation)	[241]
*miR-326*	Upregulation	Induction of apoptosis and inhibition of proliferation and invasion	*LASP1* (downregulation)	[242]
*miR-708*	Upregulation	Inhibition of proliferation, migration, and invasion	*SMAD3* (downregulation)	[243]
*miR-296-5p*	Upregulation	Inhibition of proliferation, migration, and invasion	*AKT2* (downregulation)	[244]
*miR-24-1*	Upregulation	Downregulation of c-Myc at the protein level and suppression of its O-GlcNAcylation; reduction of metastatic potential	*OGT* (downregulation)	[245]
*miR-203a-3p*	Upregulation	Inhibition of proliferation	*GPC3* (downregulation)	[246]
*miR-548aa*				
*miR-376b-3p*				
*miR-548v*				
*miR-4510*				
*miR-211-5p*	Upregulation	Inhibition of proliferation and apoptosis; enhancement of drug sensitivity	*ZEB2* (downregulation)	[247]
*miR-138*	Upregulation	Promotion of TRAIL-induced apoptosis	*ISG15* (downregulation)	[248]
*miR-592*	Upregulation	Inhibition of proliferation, migration, and invasion	*IGF-1R* (downregulation)	[249]
*miR-365*	Upregulation	Initiation of apoptosis	*Bcl-2* (downregulation)	[250]
*miR-217*	Upregulation	Suppression of proliferation, migration, and invasion; initiation apoptosis	*MTDH* (downregulation)	[251]
*miR-199a-5p*	Upregulation	Decreased cell viability and colony formation; cell cycle arrest	*CLTC* (downregulation)	[119]
*miR-185*	Upregulation	Inhibition of proliferation; G0/G1 arrest; promotion of apoptosis	*RHEB*, *RICTOR*, and *AKT1* (downregulation)	[252]
*miR-503*	Upregulation	Repression of proliferation and sensitization to anticancer drugs	*EIF4E* (downregulation)	[124]
		Inhibition of invasion and migration; repression of EMT	*PRMT1* (downregulation)	[122]
*miR-377*	Upregulation	Suppression of proliferation and induction of apoptosis	*Bcl-xL* (downregulation)	[253]
*miR-199a-3p*	Upregulation	Growth inhibition and induction of apoptosis	*ZHX1* and *PUMA* (upregulation) and *Bcl-2* (downregulation)	[166]
*miR-22*	Upregulation	Inhibition of proliferation, migration, and invasion	*CD147* (downregulation)	[254]
*miR-101*	Downregulation	Repression of TGF-*β* and CD206 in M2 cells; inhibition of macrophage-driven HCC	*DUSP1* (upregulation)	[255]
	Upregulation	Suppression of proliferation, colony formation, EMT, and angiogenesis as well as VM. Inhibition of intrahepatic and distant metastases. Synergized with doxorubicin or fluorouracil to induce apoptosis	*TGF-βR1*, *Smad2*, *SDF1*, *VE-cadherin*, *EZH2*, *COX2*, *STMN1*, and *ROCK2* (downregulation)	[128, 256, 257]
*miR-3178*	Upregulation	Inhibition of proliferation, G1 arrest, and promotion of apoptosis	*EGR3* (downregulation)	[258]
*LNA-antimiR-214*	Upregulation	Reduction in fibrosis	*miR-214* (downregulation)	[259]
*miR-190a*	Upregulation	Suppression of migration and invasion	*treRNA* (downregulation)	[260]
*miR-491*	Upregulation	Lowering of cancer stem cell-like properties; inhibition of extracellular signal-regulated kinases	*GIT-1* (downregulation)	[261]
*miR-497*	Upregulation	Inhibition of colony formation and tumor growth	*IGF-1R* (downregulation)	[262]
*miR-663*	Downregulation	Inhibition of proliferation and promotion of apoptosis	*TGFβ1* (upregulation)	[263]
*miR-20a*	Upregulation	Promotion of apoptosis; inhibition of proliferation, invasion, and migration	*CCND1* (downregulation)	[264]
*miR-148a*	Upregulation	Suppression of tumor growth and malignancy. Promotion of differentiated phenotype	*IKKα* (downregulation)	[110]
*miR-381*	Upregulation	Inhibition of proliferation, colony formation, invasion, and induction of G0/G1 arrest	*LRH-1* (downregulation)	[265]
*miR-27a-3p*	Upregulation	Inhibition of EMT, metastasis, and VM	*VE-cadherin* (downregulation)	[266]
*miR-26b-5p*	Upregulation	Suppression of Twist1-induced EMT	*SMAD1* (downregulation)	[267]
*miR-30a-5p*	Upregulation	Inhibition of proliferation, colony formation, and induction of apoptosis	*MTDH* (downregulation)	[268]
*miR-33a-3p*	Upregulation	Suppression of cellular growth and migration/invasion	*PBX3* (downregulation)	[269]
*miR-145*	Upregulation	Inhibition of activation and proliferation of hepatic stellate cells	*ZEB2* (downregulation)	[270]
*miR-1258*	Upregulation	Inhibition of proliferation, G0/G1 arrest, and induction of apoptosis	*CKS1B* (downregulation)	[271]
*miR-1299*	Upregulation	G0/G1 arrest and inhibition of proliferation	*CDK6* (downregulation)	[272]
*miR-200a*	Upregulation	Inhibition of EMT and decreased mitochondrial metabolism	*CXCL1* (downregulation)	[273]
*miR-486-5p*	Upregulation	Repression of proliferation, cellular viability, migration, and clonogenicity	*IGF-1R*, *mTOR*, *STAT3*, and *c-Myc* (downregulation)	[274]
*miR-199a-5p*	Upregulation	Inhibition of proliferation, migration/invasion, and synergized with chemotherapeutics	*E2F3* (downregulation)	[275]
*miR-1285-3p*	Upregulation	Inhibition of proliferation	*JUN* (downregulation)	[276]
*miR-449a*	Upregulation	Inhibition of motility and pulmonary metastasis; increase of epithelial markers and reduction of mesenchymal markers; reduction of Snail nuclear accumulation	*FOS* and *Met* (downregulation)	[277]
*miR-302b*	Upregulation	Sensitization to 5-FU	*MCL-1* and *DPYD* (downregulation)	[278]
*miR-143*	Downregulation	Inhibition of proliferation due to a G0/G1 arrest; induction of apoptosis	*TLR2*, *NF-κB*, *MMP-2*, *MMP-9*, *CD44*, *MMP14*, *integrin β1*, and *integrin β4* (downregulation)	[279]
*miR-324-5p*	Upregulation	Subduing invasiveness and metastatic capacity; downregulation of MMP2 and MMP9	*ETS1* and *SP1* (downregulation)	[279]
*miR-26b*	Upregulation	Inhibition of proliferation, invasion, and migration	*EphA2* (downregulation)	[280]
*miR-449*	Upregulation	Suppression of DNA replication, mitotic entry, and cellular proliferation	*SIRT1* and *SREBP-1c* (downregulation)	[281]
*miR-221*	Downregulation	Lowering of proliferation and clonogenicity; inhibition of migration/invasion; induction of G1 arrest and apoptosis	*BMF*, *BBC3*, and *ANGPTL2* (downregulation)	[282]
*miR-206*	Upregulation	Cell cycle arrest and inhibition of proliferation, invasion, and migration. Induction of apoptosis	*Notch3*, *HES1*, *Bcl-2*, and *MMP-9* (downregulation) and *p57*, *Bax*, and *cleaved caspase 3* (upregulation)	[283, 284]
*miR-148a*	Upregulation	Repression of EMT and pulmonary metastasis; increase of epithelial markers; reduction of mesenchymal markers	*Met* (downregulation)	[111]
*miR-152*	Upregulation	Inhibition of proliferation, cellular motility, and promotion of apoptosis	*TNFRF6B* (downregulation)	[285]
*miR-99a*	Upregulation	Inhibition of proliferation	*Ago2* (downregulation)	[286]
*Anti- miR-197*	Upregulation	Inhibition of migration and invasion; upregulation of CD82	*miR-197* (downregulation)	[287]
*miR-26b*	Upregulation	Sensitization of cells to doxorubicin-induced apoptosis	*TAK1* and *TAB3* (downregulation)	[288]
*let-7a*	Upregulation	Inhibition of local invasion and migration	*KRAS*, *HRAS*, and *NRAS* (downregulation)	[289]
*miR-126-3p*	Upregulation	Inhibition of migration and invasion; suppression of capillary tube formation; reduction of tumor volume and microvessel density	*LRP6* and *PIK3R2* (downregulation)	[104]
*miR-302c*	Upregulation	Attenuation of HUVECs motility; upregulation of VE-cadherin; downregulation of *β*-catenin, FSP1, and *α*-SMA; growth inhibition in cocultures	*MTDH* (downregulation)	[290]
*miR-148b*	Upregulation	Inhibition of proliferation, metastasis and angiogenesis. Improvement of chemosensitivity	*NRP1* (downregulation)	[291]
*miR-1188*	Upregulation	Inhibition of proliferation, migration, invasion, and promotion of apoptosis	*Bcl-2* and *Sp1* (downregulation)	[292]
*miR-126*	Upregulation	Inhibition of proliferation, cell cycle arrest, and induction of apoptosis	*SOX2* (downregulation)	[105]

*ADAM10*: ADAM metallopeptidase domain 10; *ADAM17*: ADAM metallopeptidase domain 17; *Ago2*: Argonaute 2; *AKT1*: AKT serine/threonine kinase 1; *AKT2*: AKT serine/threonine kinase 2; *ANGPTL2*: Angiopoietin-like 2; *ARID3B*: AT-rich interaction domain 3B; *BAD*: Bcl-2-associated agonist of cell death; *BAX*: Bcl-2-associated X; *BBC3*: Bcl-2 binding component 3; *Bcl-2*: B-cell lymphoma 2 apoptosis regulator; *Bcl-w*: Bcl-2-like protein 2; *Bcl-xL*: B-cell lymphoma extra large; *BMF*: Bcl-2 modifying factor; *CCND1*: Cyclin D1; *CCND2*: Cyclin D2; *CCNG1*: Cyclin G1; *CD133*: CD133 antigen (prominin-1); *CD147*: Cluster of differentiation 147 (Basigin); *CDK6*: cyclin-dependent kinase 6; *CKS1B*: CDC28 protein kinase regulatory subunit 1B; *CLTC*: clathrin heavy chain; *c-Myc*: Myc protooncogene; BHLH transcription factor; *COX2*: cytochrome C oxidase subunit II; *c-Raf*: Raf-1 protooncogene, serine/threonine kinase; *CXCL1*: C-X-C motif chemokine ligand 1; *DPYD*: Dihydropyrimidine dehydrogenase; *DUSP1*: Dual specificity phosphatase 1; *E2F3*: E2F transcription factor 3; *EGR3*: EGR3 early growth response 3; *EIF4E*: eukaryotic translation initiation factor 4E; *EphA2*: Ephrin receptor A2; *ETS1*: ETS protooncogene 1; *EZH2*: enhancer of zeste 2 polycomb repressive complex 2 subunit; *FOS*: Fos protooncogene, AP-1 transcription factor subunit; *FOXA1*: Forkhead box A1; *FOXO1*: Forkhead box O1; *GAB2*: GRB2-associated-binding protein 2; *GIT-1*: GIT ArfGAP 1; *GPC3*: Glypican 3; *HES1*: Hairy and enhancer of split-1; *HOXA1*: Homeobox A1; *IGF1R*: insulin-like growth factor 1 receptor; *IKKα*: Inhibitor of *κ*B kinase *α*; *IL-6*: interleukin-6; *ISG15:* interferon-stimulated gene 15; *JUN*: Jun protooncogene; AP-1 transcription factor subunit; *LASP1*: LIM and SH3 protein 1; *LIN28B*: Lin-28 homolog B; *LIN7A*: Lin-7 homolog A, crumbs cell polarity complex component; *LRH-1*: liver receptor homolog-1; *LRP6*: low-density lipoprotein receptor-related protein 6; *MCL-1*: MCL1 apoptosis regulator; *MDM2*: MDM2 protooncogene; *MET*: MET protooncogene, receptor tyrosine kinase; *MMP-2*: matrix metalloproteinase-2; *MMP-9*: matrix metalloproteinase-9, p57; *MTDH*: metadherin; *mTOR*: mammalian target of rapamycin; *NRP1*: Neuropilin-1; *OGT*: O-GlcNAc transferase; *OTUD7B*: OTU deubiquitinase 7B; *PBX3*: Pre-B-cell leukemia homeobox 3; *PDGFRβ*: Platelet-derived growth factor receptor beta; *PIK3C2α*: phosphatidylinositol-4-phosphate 3-kinase catalytic subunit type 2 alpha; *PIK3R2*: phosphoinositide-3-kinase regulatory subunit 2; *PRMT1*: protein arginine methyltransferase 1; *PUMA*: p53 upregulated modulator of apoptosis; *RHEB*: Ras homolog, mTORC1 binding; *RICTOR*: RPTOR-independent companion of MTOR, complex 2; *ROCK2*: Rho-associated coiled-coil containing protein kinase 2; *SDF1*: Stromal cell-derived factor 1; *SETDB1*: SET domain bifurcated histone lysine methyltransferase 1; *SIAH1*: Siah E3 ubiquitin protein ligase 1; *SIRT1*: Sirtuin 1; *SIX1*: SIX homeobox 1; *SMAD1*: SMAD family member 1; *SMAD2*: SMAD family member 2; *SMAD3*: SMAD family member 3; *SOX2*: sex-determining region Y-box 2; *SP1*: Transcription factor Sp1 (specificity protein 1); *SREBP-1c*: Sterol regulatory element binding protein-1c; *STAT3*: signal transducer and activator of transcription 3; *STMN1*: Stathmin 1; *TAB3*: TGF beta-activated kinase binding protein 3; *TAK1*: Transforming growth factor beta-activated kinase 1; *TGFβ1*: Transforming growth factor beta 1; *TGF-βR1*: Transforming growth factor beta receptor 1; *TNFRF6B*: Tumor necrosis factor receptor super family 6B; *TNIP2*: TNFAIP3 interacting protein 2; *VEGFR*: vascular endothelial growth factor receptor; *ZEB2*: Zinc finger E-box binding homeobox 2; *ZHX1*: Zinc fingers and homeobox 1.

**Table 5:** Dysregulated long noncoding RNAs (lncRNAs) in HCC. Long noncoding RNAs are shown with the trend of dysregulation associated with HCC. As is evident, the majority of dysregulated lncRNAs follow an upward tendency. Also evident is the involvement of lncRNA-mediated miRNA sponging in producing the oncogenic molecular phenotypes.

lncRNA	Expression in HCC	Effect of dysregulation	Ref.
*91H*	Upregulated	Promoting tumor growth and metastasis; upregulation of *IGF2*, H3K4me3, and H3K27me3 at the P3 and P4 promoters	[208]
*AC006262.5*	Upregulated	Inhibition of *miR-7855-5p* and upregulation of *BPY2C*	[200]
*AC092171.4*	Upregulated	Inhibition of *miR-1271* and upregulation of *GRB2*	[182]
*ANCR*	Upregulated	Enhanced proliferation and EMT; upregulation of *HNRNPA1* through *miR-140-3p* sponging	[151]
*ANRIL*	Upregulated	Inhibition of *miR-384* and upregulation of *STAT3*	[214]
*ASMTL-AS1*	Upregulated	Upregulation of *NLK* and activation of *YAP* signaling via *miR-342-3p* sponging	[293]
*CASC2c*	Downregulated	Activation of *ERK1/2* and *Wnt/β-catenin* signaling	[201]
*CASC15*	Upregulated	Activation of *Wnt/β-catenin* signaling via upregulation of *SOX4*	[196]
*CRNDE*	Upregulated	Inhibition of the *Hippo* pathway	[210]
*CTBP1-AS2*	Upregulated	Sponging of *miR-195-5p* and enhancing *CEP55* expression	[198]
*DANCR*	Upregulated	Enhanced cell proliferation, colony formation, and autophagy; upregulation of *ATG7* and suppression of *miR-222-3p*	[188]
*DDX11-AS1*	Upregulated	Inhibition of *LATS2* expression via *EZH2* and *DNMT1*	[203, 204, 207]
*DUXAP8*	Upregulated	Enhanced cell proliferation and EMT; *miR-422a* sponging and upregulation of *PDK2*	[147]
*FENDRR*	Downregulated	Downregulation of *GPC3*	[168]
*FOXD2-AS1*	Upregulated	*miR-206* sponging and enhanced *MAP3K1* signaling	[174]
*FOXD3-AS1*	Upregulated	*miR-335* sponging and upregulation of *RICTOR*	[159]
*GAS8-AS1*	Downregulated	Attenuated *GAS8* transcription RNA polymerase II activity	[157]
*H19*	Upregulated	Amelioration of resistance to sorafenib and upregulation of *miR-675*	[177]
*HAND2-AS1*	Downregulated	Enhanced proliferation; upregulation of *miR-300* and inhibition of *SOCS5*	[155]
*HBVPTPAP*	Upregulated	Activation of *JAK/STAT* signaling	[186]
*HCG18*	Upregulated	Upregulation of *CENPM* via sponging of *miR-214-3p*	[180]
*HEIH*	Downregulated	Suppression of cell proliferation and metastasis; upregulation of *miR-199a-3p*	[169]
*HLNC1*	Upregulated	Destabilization of *USP49*	[183]
*HOTAIR*	Upregulated	Downregulation of *c-Met* and *miR-34a*	[178, 184]
*HOXA11-AS*	Upregulated	Downregulation of *miR-506-3p* and *Slug*	[191]
*KCNQ1OT1*	Upregulated	Upregulation of *ACER3* via sponging of *miR-146a-5p*; enhanced sorafenib resistance and *PD-L1*-mediated immune escape via *miR-506* sponging	[197, 205]
*LALR1*	Upregulated	Anaplasia and distant metastases; upregulation of *SNORD72*	[154]
*LEF1-AS1*	Upregulated	Enhancement of tumor growth and chemoresistance; inhibition of *miR-10a-5p* and upregulation of *MSI*, *CDCA7*, and *EZH2*	[162, 194]
*LINC00160*	Upregulated	Inhibition of miR-132 and elevated levels of PIK3R3	[144]
*LINC00174*	Upregulated	Enhanced proliferation and metastasis and decreased apoptosis; sponging of *miR-320* and upregulation of *S100A10*	[152]
*LINC00467*	Downregulated	Sponging of miR-9-5a and consequent upregulation of *PPARA*	[140]
	Upregulated	Posttranscriptional inhibition of *NR4A3*	[153]
*LINC00662*	Upregulated	Genome-wide hypomethylation; modulation of *MAT1A/SAM* and *AHCY/SAH* interactions, leading to reduced *SAM* and increased *SAH*	[294]
*LINC00668*	Upregulated	Promoting cell proliferation and EMT; sponging of *miR-532-5p* and consequent upregulation of *YY1*	[161]
*LINC00978*	Upregulated	Inhibition of *p21* and *E-cadherin* via *EZH2*-mediated silencing	[211]
*LINC01224*	Upregulated	Inhibition of *miR-330-5p* and consequent upregulation of *CHEK1*	[212]
*LINC01278*	Upregulated	Promoting metastasis; inhibition of *miR-1258*	[164]
*LINC01296*	Upregulated	Positive regulation of the *miR-26a/PTEN* axis	[137]
*LINC01419*	Upregulated	Histone methylation of the *RECK* promoter via *EZH2*	[173]
*Linc-GALH*	Downregulated	Upregulation of *Gankyrin*	[190]
*lncARSR*	Upregulated	Reduction of *YAP1* phosphorylation and activation of *IRS2/AKT* signaling	[156]
*lncRNA-POIR*	Upregulated	Enhanced EMT and sorafenib resistance; sponging of *miR-182-5p*	[202]
*MALAT1*	Upregulated	Tumor progression and doxorubicin resistance; *miR-3129-5p* sponging, upregulation of *β-catenin*	[199, 209]
*MFI2-AS1*	Upregulated	Improved proliferation and metastasis; sponging of *miR-134* and upregulation of *FOXM1*	[142]
*MINCR*	Upregulated	Enhanced proliferation and inhibition of apoptosis; downregulation of *miRNA-107*	[150]
*miR503HG*	Downregulated	Enhanced invasion and metastasis; activation of *NF-κB* signaling	[141]
*MSC-AS1*	Upregulated	Promoting cell proliferation and colony formation; suppression of *PGK1*	[172]
*MT1JP*	Downregulated	Repression of tumor growth; decreased *AKT* expression	[170]
*NEAT1*	Upregulated	Upregulation of *WEE1* through *miR-101-3p* sponging; inhibition of *miR-129-5p*	[129, 138]
*OIP5-AS1*	Upregulated	Promoting cell proliferation, migration and angiogenesis. Inhibition of apoptosis; inhibition of the *miR-26a-3p* and *miR-3163*	[163, 171]
*OTUD6B-AS1*	Upregulated	Enhanced proliferation and colony formation; sponging of *miR-664b-3p*	[181]
*PICSAR*	Upregulated	Enhanced proliferation and colony formation; sponging of *miR-588*	[189]
*RHPN1-AS1*	Upregulated	Promoting proliferation, migration and invasion; suppression of *miR-485-5p*	[165]
*RUNX1-IT1*	Downregulated	Desponging of *miR-632* and activation of *WNT/β-catenin* pathway	[148]
*RUSC1-AS1*	Upregulated	Enhanced proliferation and reduced apoptosis; *miR-7-5p* sponging and upregulation of *NOTCH3*	[185]
*SLC2A1-AS1*	Downregulated	Suppression of glycolysis in HCC cells; downregulation of *GLUT1*	[158]
*SNAI3-AS1*	Upregulated	Promoting proliferation and metastasis; activation of *PEG10* sponging *miR-27-3p* and *miR-34a-5p*	[195]
*SNHG1*	Upregulated	Enhanced tumor progression and metastasis; sponging of *miR-377-3p*	[149]
*SNHG5*	Upregulated	Sponging of *miR-26a-5p* and upregulation of the downstream target, *RNF38*	[160]
*SNHG14*	Upregulated	Inhibition of *miR-656-3p*, promotion of migration and invasion	[176, 187]
*SOX2OT*	Upregulated	Promoting the Warburg effect and metastasis; upregulation of *PKM2* via *miR-122-5p* inhibition	[167]
*SUMO1P3*	Upregulated	Enhanced cell proliferation and lymph node metastasis; *miR-320a* sponging and activation of *Wnt/β-catenin* signaling	[146]
*TCL6*	Downregulated	Activation of PI3K/AKT signaling via upregulation of *miR-106a-5p*	[145]
*TMPO-AS1*	Upregulated	Promoting proliferation, migration, and invasion; *miR-329-3p* sponging	[166]
*TUG1*	Upregulated	Negative regulation of *miR-137* and *AKT2* and promoting EMT	[175]
*UBE2R2-AS1*	Upregulated	*miR-302b* sponging and upregulation of *EGFR*	[192]
*UC001kfo*	Upregulated	Enhanced proliferation, macrovascular invasion, and EMT; upregulation of *α-SMA*	[179]
*ZFAS1*	Upregulated	Enhanced proliferation; *miR-193a-3p* suppression	[213]
*ZFPM2-AS1*	Upregulated	Enhanced proliferation, migration, and invasion; inhibition of *miR-139*	[193]
*ZNF281*	Upregulated	Promoting migration and invasion; downregulation of *miR-539*	[143]

*ACER3*: Alkaline Ceramidase 3; *AHCY*: Adenosylhomocysteinase; *AKT*: Protein kinase B; *ATG7*: Autophagy-related 7; *BPY2C*: Basic Charge Y-Linked 2C; *CDCA7*: Cell Division Cycle-Associated 7; *CENPM*: Centromere Protein M; *CEP55*: Centrosomal Protein 55; *CHEK1*: checkpoint kinase 1; *c-Met*: Tyrosine-protein kinase *Met*; *DNMT1*: DNA methyltransferase 1; *EGFR*: epidermal growth factor receptor; *ERK*: extracellular signal-regulated kinase; *EZH2*: enhancer of zeste homolog 2; *FOXM1*: Forkhead box protein M1; *GAS8*: growth arrest-specific 8; *GLUT1*: Glucose transporter 1; *GPC3*: Glypican 3; *GRB2*: growth factor receptor-bound protein 2; *HNRNPA1*: heterogeneous nuclear ribonucleoprotein A1; *IGF2*: insulin-like growth factor 2; *IRS2*: *insulin receptor substrate 2*; *JAK*: Janus kinase; *LATS2*: large tumor suppressor 2; *MAP3K1*: mitogen-activated protein kinase 1; *MAT1A*: Methionine Adenosyltransferase 1A; *MSI*: RNA-binding protein Musashi; *NF-κB*: nuclear factor kappa-light-chain-enhancer of activated B cells; *NLK*: Nemo-Like Kinase; *NOTCH3*: Notch Receptor 3; *NR4A3*: Nuclear Receptor Subfamily 4 Group A Member 3; *p21*: cyclin-dependent kinase inhibitor 1; *PDK2*: Pyruvate dehydrogenase kinase isoform 2; *PD-L1*: Programmed death-ligand 1; *PEG10*: Paternally Expressed 10; *PGK1*: Phosphoglycerate Kinase 1; *PI3K*: Phosphoinositide 3-kinase; *PIK3R3*: Phosphoinositide-3-Kinase Regulatory Subunit 3; *PKM2*: Pyruvate kinase muscle isozyme; *PPARA*: peroxisome proliferator-activated receptor alpha; *PTEN*: Phosphatase and tensin homolog; *RECK*: Reversion-inducing-cysteine-rich protein with kazal motifs; *RICTOR*: Rapamycin-insensitive companion of mammalian target of rapamycin; *RNF38*: Ring Finger Protein 38; *S100A10*: S100 Calcium Binding Protein A10; *SAH*: S-adenosyl homocysteine; *SAM*: S-adenosyl-L-methionine; *SNORD72*: Small Nucleolar RNA, C/D Box 72; *SOCS5*: Suppressor of cytokine signaling 5; *SOX4*: SRY-Box Transcription Factor 4; *STAT3*: signal transducer and activator of transcription 3; *USP49*: Ubiquitin-Specific Peptidase 49; *WEE1*: WEE1 G2 Checkpoint Kinase; *YAP*/*YAP1*: Yes-associated protein 1; *YY1*: Yin Yang 1; *α-SMA*: *alpha*-smooth muscle actin.
